# Microplastic deposition velocity in streams follows patterns for naturally occurring allochthonous particles

**DOI:** 10.1038/s41598-019-40126-3

**Published:** 2019-03-06

**Authors:** Timothy J. Hoellein, Arial J. Shogren, Jennifer L. Tank, Paul Risteca, John J. Kelly

**Affiliations:** 1 Dept. Biology, Loyola Univ. Chicago, 1032 W Sheridan Rd, Chicago, IL 60660 USA; 2 Dept. Biological Sciences, Univ. of Notre Dame, Notre Dame, IN 46556 USA; 3 Present Address: Dept. Earth and Env. Sci., Michigan State Univ., 306 Natural Sci. Bldg, East Lansing, MI 48824 USA

## Abstract

Accumulation of plastic litter is accelerating worldwide. Rivers are a source of microplastic (i.e., particles <5 mm) to oceans, but few measurements of microplastic retention in rivers exist. We adapted spiraling metrics used to measure particulate organic matter transport to quantify microplastic deposition using an outdoor experimental stream. We conducted replicated pulse releases of three common microplastics: polypropylene pellets, polystyrene fragments, and acrylic fibers, repeating measurements using particles with and without biofilms. Depositional velocity (v_dep_; mm/s) patterns followed expectations based on density and biofilm ‘stickiness’, where v_dep_ was highest for fragments, intermediate for fibers, and lowest for pellets, with biofilm colonization generally increasing v_dep_. Comparing microplastic v_dep_ to values for natural particles (e.g., fine and coarse particulate organic matter) showed that particle diameter was positively related to v_dep_ and negatively related to the ratio of v_dep_ to settling velocity (i.e., sinking rate in standing water). Thus, microplastic v_dep_ in rivers can be quantified with the same methods and follows the same patterns as natural particles. These data are the first measurements of microplastic deposition in rivers, and directly inform models of microplastic transport at the landscape scale, making a key contribution to research on the global ecology of plastic waste.

## Introduction

Plastic is a critical component of modern economies worldwide, and the production of plastic has accelerated since its industrialization in the mid-1900s^1^. Generation of plastic waste has accelerated concurrent with rates of production, and plastic litter is found in environments throughout the world, including oceans^2,3^, freshwaters^4,5^, soils^6,7^, and the atmosphere^8^. Microplastic particles (<5 mm) accumulate in the environment as primary microplastic (e.g., small pieces used for manufacturing of plastic goods, and abrasives in personal care products and cleaners), or secondary microplastic that is generated via fragmentation of larger plastic items (e.g., fibers from synthetic textiles or amorphous plastic particles)^9^. Microplastic particles are an emerging contaminant of concern in aquatic ecosystems due to their biological and chemical interactions, as well as the general resistance of plastic polymers to biodegradation^10,11^.

Although streams and rivers are suspected to be a major source of marine plastic, empirical measurements of microplastic inputs, deposition, accumulation, and downstream transport in flowing waters are uncommon. Importantly, streams and rivers can act as both conduits and accumulators of microplastics^5,12^, as microplastics are found in higher abundance in benthic sediments compared to the water column, suggesting sediments are a sink for microplastic^12–14^. However, flowing waters are also sources of microplastic to downstream environments when benthic microplastic is mobilized during disturbance or flooding^15^, yet rates of microplastic retention, via deposition from the water column to the benthos, have not yet been quantified for flowing waters. Accurate measurements of microplastic deposition rates are needed in order to understand the role of streams and rivers in retaining and transporting plastic waste across the landscape, and ultimately for constraining global plastic budgets^16^.

Stream ecosystems are well-studied in regards to the movement, breakdown, and transport of naturally occurring particles such as coarse particulate organic matter (CPOM; i.e., leaf litter), fine particulate organic matter (FPOM), and wood^17–19^. Plastic litter is a novel and recalcitrant form of allochthonous carbon in lotic ecosystems. Thus, its movement can be quantified using the same methods as naturally occurring particles, whereby transport and retention of particles is measured using ‘spiraling metrics’^20–22^, which integrate the rates of downstream transport, deposition, breakdown, and turnover^23,24^. While streams are net sources of particulates to downstream environments (e.g., larger rivers and coastal marine environment), their capacity to retain and process particles during each step of the spiral is an important component of global carbon and nutrient budgets^25,26^. Spiraling metrics have not yet been applied to plastic particles, but will be critical for estimating current and future global measurements of plastic distribution and movement.

In marine ecosystems, microplastic movement is affected by currents, biofilm colonization (i.e., heterogeneous mixture of bacteria, algae, and fungi attached to a solid surface), ingestion, and degradation via ultraviolet light and microbial breakdown^16,27^. Although deposition and transport rates have not been measured in flowing waters, we predict that physical and biological processes will influence microplastic retention in streams in the same fashion as for other fine particles. For example, microplastic collected in streams was colonized by distinct bacterial communities relative to natural particles, exhibiting a high abundance of gastrointestinal pathogens and potential degraders of plastic polymers^28^. Downstream movement of plastic may disperse microbial biofilm constituents in novel ways, or eventually break down plastic compounds^10^, and as such, biofilm colonization likely affects microplastic deposition and flocculation, which deserves quantitative examination.

Our objective was to conduct direct measurements of deposition and transport of primary and secondary microplastics in a stream, using different particle types, with and without biofilm colonization. We predicted we would measure the highest deposition of microplastic particles for the most-dense particles (i.e., acrylic fibers), and the lowest deposition for the more buoyant microplastic pellets with the lowest surface area:volume ratio. We also expected that biofilm coverage would reduce particle transport, relative to ‘virgin’, un-colonized plastic, due to enhanced ‘stickiness’ resulting from the polysaccharide matrix typical of biofilms which imparts increased surface complexity^29^.

## Methods

### Study site

We conducted additions of microplastic in an experimental stream at the University of Notre Dame’s Linked Experimental Ecosystem Facility (ND-LEEF) in South Bend, IN, USA (https://environmentalchange.nd.edu/resources/nd-leef/). Replicated streams at ND-LEEF have been successfully used to study controls on the movement of solutes and particles in flowing waters^30–32^. Our chosen study stream has a mean width (±SE) of 48 cm (±1.8) and depth of 3.7 cm (±0.2), and is lined with a substrate of uniformly-sized pea gravel (D_50_ = 0.5 cm)^33^. Discharge (Q) was maintained at constant of 1.45 L/s with ball valve at the upstream end of the stream reach, releasing water from an adjacent, low-nutrient, groundwater-fed reservoir. Riparian vegetation consisted of mixed native grasses and forbes; any adjacent prairie vegetation that had fallen into the stream was removed prior to the microplastic addition.

### Pulse release of microplastic

We conducted pulse releases for three types of microplastic commonly found in urban streams and rivers^12,28^. Polypropylene pellets were purchased directly from the supplier (diameter 3 mm; Engineering Laboratories, Inc., Oakland, NJ, USA). Polystyrene fragments were generated in the lab by cutting disposable cups into pieces (Neon assorted 10 oz tumblers, NorthWest Enterprizes, Elk Grove Village, IL, USA), fragmenting the pieces in a grinder (Spice and Nut Grinder SG10, Cuisinart, East Windsor NJ, USA), and sorting the fragments between 1–2 mm sieves. We saved the 1–2 mm size class for experimental additions, and discarded the smaller and larger fragments. Acrylic fibers were generated in the lab from yarn (Colorations, Discount School Supply, Carol Stream, IL, USA), using a razorblade to cut it into 1–2 mm fragments. Fibers and fragments were in a typical size range for streams in the region, while pellets were slightly larger than commonly encountered^13,28^ to facilitate our imaging methods. We placed prepared microplastic into pre-cleaned, 500 ml glass bottles. We conducted a total of 18 releases (N = 6 per microplastic type), where half of the releases were subject to biofilm colonization. We facilitated biofilm colonization by collecting benthic sediment and overlying water from the North Branch Chicago River^34^, placing ~50 ml of sediment + water slurry in bottles with microplastic (N = 9), and placing them on a shaking incubator table for 7 days (37 °C, 200 rpm). We removed several replicate particles (5–15) from each of the biofilm and non-biofilm containers, freezing them at −20 °C until measurement of bacterial DNA abundance via quantitative PCR (see below).

We conducted experimental microplastic releases in three separate, 20 m reaches of the experimental stream. Prior to conducting the microplastic releases, we completed a pulse addition of dissolved salt (30 g NaCl in 400 mL water) to measure travel time to each collection station. We then released microplastic in a pulse addition (along with reference salt solution), with one type of plastic particle, at the top of each 20 m reach, and collected water column samples at 5, 10, and 15 m downstream. To measure fragments and pellets, we used cameras to take pictures of the stream surface every 5–10 seconds (iPhone, Cupertino, CA, USA; N = 20–35 per collection site) by three separate researchers, and framed each image with rulers set up over the stream to establish a permanent 40 × 50 cm ‘reference window’ (Supplemental Fig. 1). We recorded sampling times using stopwatches at each station, placed in the upper left corner of the reference frame in each image. Fibers are not visible to the naked eye, so we collected water samples in 160 ml specimen containers every 5–15 sec following addition.

We conducted six releases in each 20 m reach of the experimental stream, one for each particle type and biofilm colonization status combination per reach. We started in the most downstream reach, and moved upstream for each set of six releases to avoid any contamination from previous releases. We used different colored fibers and fragments to distinguish the particles that had biofilm colonization from those that did not. All pellets were white, however, and we note some particles from one release might have been counted in the subsequent pellet release. We observed little cross contamination between releases in the same reach for the fibers and fragments (which were different colors), so we assume this effect was minimal for pellets. After all pulse releases were completed, we measured stream width at 5 locations in each 20 m reach, and measured water depth at 4 locations across each width transect (N = 20). We limited plastic dispersal by conducting all releases in a single experimental stream, and after the final release, we placed two fine mesh nets at the end of the stream, and thoroughly agitated the streambed to move all particles into the net.

We measured microplastic abundance from images and water samples in the laboratory, counting pellets and fragments in each image, individually. We only counted particles at the time of their first appearance. We note this involved occasional speculation about particles being newly appeared at the time of each image or if they moved within the ‘frame’ of the collection site between pictures taken in sequence. Most often, when the same particle appeared in two sequential pictures, it remained in that spot for the duration of the series of images. For the water samples containing fibers, we poured the water from the container into a filter stand with a glass fiber filter (0.7 μm nominal pore size, Whatman, Piscataway, NJ, USA), measured conductivity (YSI Model 3010, Yellow Springs, OH, USA), and then vacuum-filtered the sample. We carefully rinsed the conductivity probe over the filter, and rinsed the filter stand between samples with deionized water. We placed filters in metal tins and covered them with aluminum foil until we counted particles under a dissecting scope at 25–50 × magnification. Contamination of microplastic fibers in laboratory conditions is common^5,13^, however, we found contamination to be minimal in this study, and could easily be excluded from counts due to the uniformity in color and size of the added microplastic. We used conductivity measurements from the specimen containers as a tracer to calculate stream discharge via dilution of the conductivity of the release solution^35^, which we then used in calculation of subsequent deposition rates.

We used standard methods for measuring transport and retention of organic matter and solutes to quantify microplastic deposition^19,36,37^. First, we plotted the number of particles at each station at each time, integrating under the resulting curve, to quantify the number of particles that moved by each sampling station. We then divided particle number by the integrated value for the conservative tracer at the same site and time, plotting the natural log of the ratio of microplastic/conductivity vs distance downstream (5, 10, and 15 m; Supplemental Fig. 2). The inverse of the slope of the line represents a transport length (S_w;_; m), or the average distance a particle traveled before being removed from the water column via deposition. We then calculated a depositional velocity (v_dep_; m/s) for each release, scaling for stream discharge and width, using the following equation:

$${{\rm{v}}}_{{\rm{dep}}}={\rm{Q}}/{{\rm{S}}}_{{\rm{w}}}/{\rm{w}}$$

where discharge (Q; m^3^/s) and average width (w; m) were measured directly for each stream reach. This value can be considered an effective vertical transfer coefficient of particles from the water column to the benthic surface in field conditions, or the effective velocity that particles leave the water column, scaled to the reach of stream under study. The value is specifically designed to allow for a comparison of particle transport length (S_w_) across streams of variable discharge and size^20,38^.

As a comparative metric, we measured sinking velocity (v_fall_; the settling rate of a particle in quiescent water) for fragments and estimated v_fall_ of fibers from literature values. We could not measure v_fall_ for pellets as the density (ρ_p_) of polypropylene (0.946 g/cm^3^) was less than stream water, causing the material to float, even after biofilm colonization. For fragments, we measured v_fall_ in 8 × 8 cm glass vessel filled with 14.3 cm of deionized water at 18.5 °C. We randomly selected individual fragments from a batch of non-biofilm colonized fragments prepared for the stream plastic addition but not used. Using forceps, we placed an individual fragment just below the surface of the water, and carefully timed its descent to the bottom. We repeated this for 50 fragments which generated a mean (±standard deviation) v_fall_ of 15.6 (±1.8) mm/s. We acknowledge the laboratory water temperature was higher than *in situ* temperature for vdep (18.5 °C and 10 °C, respectively). For fiber v_fall_, we used published values of direct measurements of v_fall_ for fibers made of monofilament fishing line of varying length and width^39^ where monofilament ρ_p_ = 1.13–1.17 g/cm^3^, r = 0.15 mm, and length = 1.5 mm, to estimate v_fall_ of acrylic fibers at 6.0 mm/s (acrylic particles ρ_p_ = 1.17 g/cm^3^, r = ~0.1 mm, length = 1.5 mm).

### Biofilm colonization

We used quantitative polymerase chain reaction (qPCR) of 16 s rRNA genes as an indicator of bacterial abundance on microplastic samples, on three replicate samples of each particle type (pellets, fragments, and fibers) and treatment (control and biofilm-colonized). We extracted community genomic DNA from each sample using the MoBio Power Biofilm DNA Isolation Kit (MoBio, Carlsbad, California, USA) and stored samples at −20 °C. We also ran a kit control by running the DNA Isolation Kit with no sample; the extract from this kit control was run through the qPCR assay in parallel with the samples, and 16S rRNA gene copy numbers from the kit control were subtracted from all samples. To provide a standard for qPCR, we extracted genomic DNA from frozen cell pellets of *Pseudomonas aeruginosa* PAO1 using the MoBio Microbial DNA Isolation Kit (MoBio); we determined the concentration of *P. aeruginosa* DNA based on UV absorbance using a FLUOstar Omega microplate reader (BMG Labtech, Cary, NC, USA). The genome of *P. aeruginosa* is 6,264,404 bp (based on NCBI BioProject PRJNA331) and includes 4 copies of the 16S rRNA gene (based on University of Michigan rRNA Database rrndb.umms.med.umich.edu). We used these data to calculate the number of 16S rRNA gene copies in our *P. aeruginosa* DNA, and to establish a set of standards for qPCR ranging from ~900 to 9 million copies.

We ran qPCR reactions using an MJ Research DNA Engine Opticon1 thermal cycler equipped with Opticon Monitor software version 3.1 (Biorad, Hercules, CA, USA). Conditions for all qPCR reactions were as follows: 12.5 μl QuantiTect SYBR Green PCR Master Mix (Qiagen, Valencia, CA, USA), 0.5 μM final concentration of each primer, 5 μl template, and water were added to a final 25 μl volume. qPCR was carried out using primers 515 F and 806R^40^. All reactions were performed in low-profile 0.2 mL white strip tubes with optical ultra-clear strip caps (Bio-Rad). Thermal cycling was as follows: initial denaturation at 94 °C for 15 min, 40 cycles of denaturation at 94 °C for 15 sec, primer annealing at 56.4 °C for 30 sec, extension at 72 °C for 90 sec, hold at 78 °C for 1 sec, and plate read. Finally, a melting curve was run from 50 to 95 °C with a read every 1 °C and a hold of 1 sec between reads. Specificity of qPCR reactions was confirmed by melting curve analysis and agarose gel electrophoresis. Sample quantification was based on comparison to standard curves using Opticon Monitor software version 3.1 (Biorad).

### Comparison of microplastic deposition rates to literature values

We conducted two literature comparisons to place microplastic v_dep_ results in context of similar measurements for other particles made in flowing waters. First, we assembled results for data using a literature search for publications that provided empirically measured v_dep_ for fine particulate organic matter (FPOM), coarse particulate organic matter (CPOM), and other particle analogs used for FPOM (e.g., pollen, glass beads, brewer’s yeast; Supplemental Table 2). Where needed, we estimated particle area from the published diameter using equations for a sphere (applied to FPOM, pollen, yeast) or cylinders (for fibers, sticks). To compare the ratio of v_dep_/v_fall_ with particle diameter among many different particle types, we began with a data synthesis by Georgian *et al*.^17^, and added results from Hunken and Mutz^41^, Cushing *et al*.^38^, and microplastic v_dep_/v_fall_ values for fragments and fibers.

### Data analysis

We used two-way ANOVA to quantify the differences in S_w_, v_dep_, and 16S rRNA gene copies by particle type and biofilm colonization status. We used natural log transformations of S_w_ and v_dep_ values to meet the assumption of normal distribution. We used simple linear regression to determine the relationship between particle diameter and area with v_dep_, as well as particle diameter and v_fall_/v_dep_ for microplastic combined with the log-transformed values from the literature for other particles^17,39,42^. We completed all statistical analyses using SYSTAT 13 (Systat Inc, Chicago, IL, USA).

## Results

**Figure 1:**
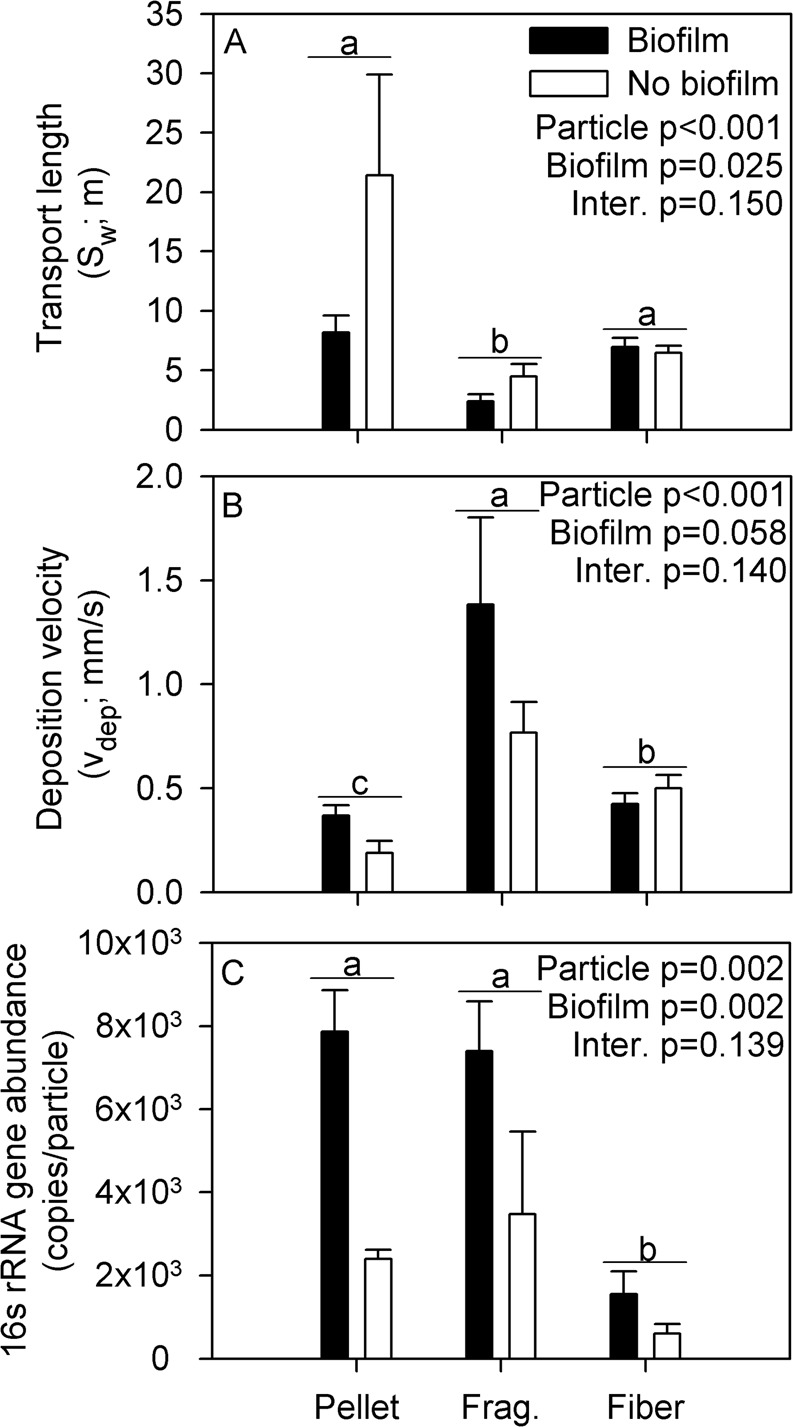
Mean (±SE) values for (**A**) transport length (S_w_) and (**B**) deposition velocity (v_dep_) for microplastic pellets, fragments (frag), and fibers, separated by biofilm colonization status. (**C**) The number of copies for 16S rRNA genes was an indicator of bacteria abundance on particles. Results from two-way ANOVA comparing values by particle type and biofilm colonization are shown. Small letters indicate differences among particle types following a significant ANOVA, as shown by Tukey’s post-hoc test.

Particle transport length (S_w_) and depositional velocity (v_dep_) showed significant differences between particle type and biofilm colonization. Pellets and fibers had longer S_w_ than fragments (two-way ANOVA, p < 0.001; Fig. 1A). Similarly, v_dep_ was highest for fragments, intermediate for fibers, and lowest for pellets (two-way ANOVA, p < 0.001; Fig. 1B). For both S_w_ and v_dep_, particles with biofilm colonization showed greater retention than particles without (two-way ANOVA, S_w_ p = 0.025_,_ v_dep_ p = 0.058; Fig. 1A,B). The interaction between biofilm colonization and particle type was not significant for S_w_ or v_dep_ (two-way ANOVA, p = 0.150 and 0.140, respectively). Finally, we confirmed greater abundance of 16 s RNA gene copies on biofilm-colonized particles using qPCR (two-way ANOVA, p = 0.002; Fig. 1C). Fibers had fewer gene copies than pellets and fragments (two-way ANOVA, p = 0.002), and the interaction term was not significant (two-way ANOVA, p = 0.139; Fig. 1C).

**Figure 2:**
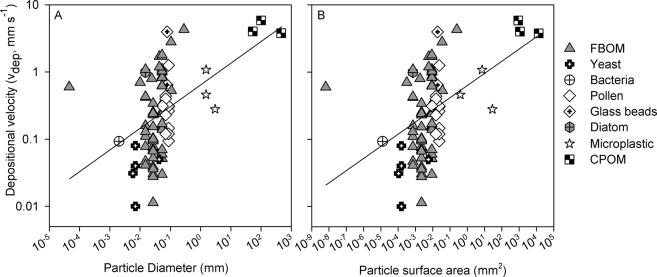
Meta-analysis of deposition velocity (v_dep_) in streams according to (**A**) particle diameter, and (**B**) particle surface area across many different particle types, including fine particulate organic matter (FPOM)^17,18,38,41,45,53,56,57^, bacteria^58^, yeast^59^, Shogren *et al*. Unpublished data, diatoms^18^, pollen^17,19^, Shogren *et al*., unpublished data^23,46^, glass beads^19,46^, moss spores (*Lycopodium* sp.)^60^, coarse particulate organic matter (CPOM; deciduous leaves, pine needles, and sticks)^61^, and microplastic (this study). For all data see Supplemental Table 2.

Comparing v_dep_ for microplastic to literature values for other particles suggested that the physical properties of microplastic particles helped explain variation in v_dep_ values. With all particle types combined, v_dep_ and particle diameter showed a significant positive relationship (r^2^ = 0.20, p < 0.001; Fig. 2A). The same relationship was shown for particle surface area, (r^2^ = 0.19, p < 0.001; Fig. 2B). The largest particles, CPOM, had a strong impact on the regression results, therefore, inferences about the nature and strength of this relationship should be viewed with the following context. With the CPOM data removed, the relationship between v_dep_ and particle diameter has less explanatory power (r^2^ = 0.07, p = 0.011), with the same result for v_dep_ and particle surface area (r^2^ = 0.07, p = 0.011). We note that v_dep_ measurements were not evenly distributed across size classes, with an abundance of measurements for particles between 0.01–0.1 mm in diameter, and relatively few measurements for particles >1 mm in diameter (Fig. 2A). Finally, we also note that the variation in v_dep_ for particles in the 0.01–0.1 mm range was high (Fig. 2A,B).

**Figure 3:**
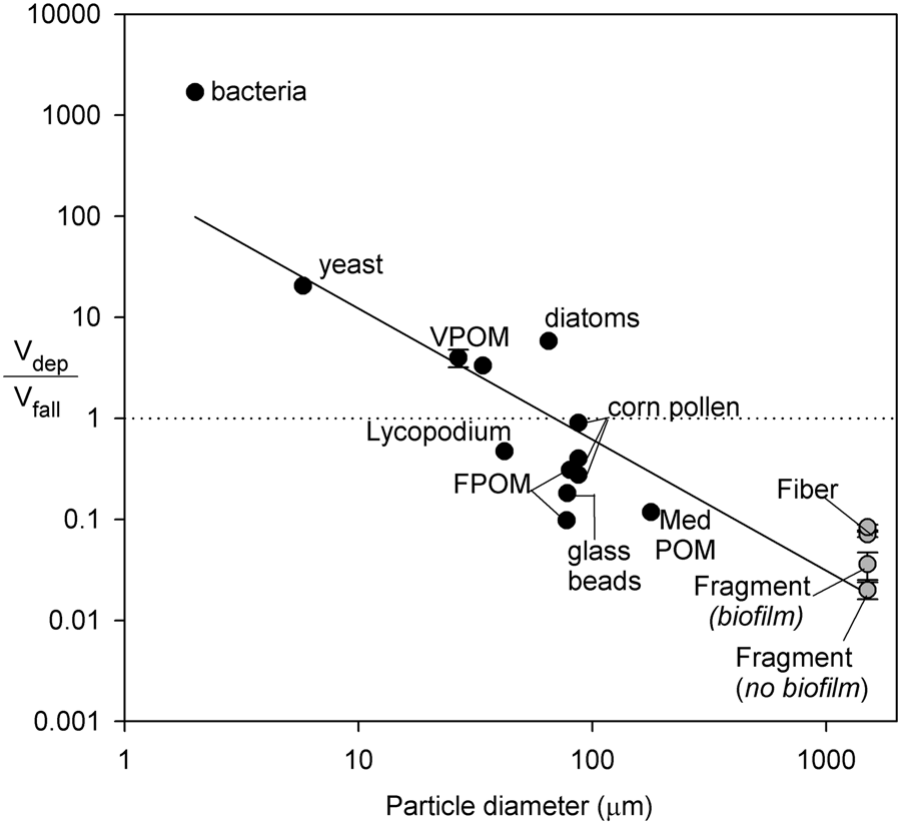
The synthesis of particle transport presented by Georgian *et al*.^17^ compared the ratio of deposition velocity (v_dep_) to settling velocity (v_fall_) relative to particle diameter (log transformed), for data collected in flowing waters. We added data for microplastic fragments and fibers collected in this study, with and without biofilms, as well as data from Cushing *et al*.^38^ and Hunken and Mutz^41^. Abbreviations: VPOM = very fine particulate organic matter, FPOM = fine particulate organic matter, Med POM = medium particulate organic matter, Lycopodium = bryophyte spores. The dotted horizontal line indicates v_dep_/v_fall_ = 1, where deposition velocity is equal to expectations from settling velocity.

We added microplastic v_dep_/v_fall_ results to a previous synthesis by Georgian *et al*.^17^, and found a significant negative relationship between v_dep_/v_fall_ and particle diameter among particle types (r^2^ = 0.78, p < 0.001, y = −1.30x +2.38; Fig. 3). The regression showed very similar results with microplastic excluded (r^2^ = 0.85, p < 0.001, y = −2.0x +3.40). For particles where the v_dep_/v_fall_ ratio is ~1 (i.e., dashed line Fig. 3), v_dep_ followed the expectations of deposition rate from gravity alone, occurring in the mid-range of the x-axis values (~50–500 µm; Fig. 3). Where v_dep_/v_fall_ ratio was ≫1, particles were retained at a higher rate than expected by gravity alone (i.e., particles <50 µm; Fig. 3). In contrast, larger particles (i.e., diameter >500 µm) showed v_dep_/v_fall_ ratios ≪1, suggesting hydrologic factors such as momentum and resuspension move particles downstream to a greater degree than expected by gravitational settling. Overall, comparing particle diameter with v_dep_/v_fall_ explained more variation in the data than the regression between particle diameter and v_dep_ alone (Fig. 2). However, we note that we excluded some larger particles from the v_dep_/v_fall_ analysis, that were used in the v_dep_ analysis, because their buoyancy results in v_fall_ at 0 mm/s (i.e., leaves, sticks, pine needles, and pellets).

## Discussion

Understanding the fate, transport, and biological interactions of plastic in aquatic ecosystems at a global scale requires measuring rates of plastic input, retention, and export in streams and rivers, as well as the identification of the environmental factors that control these processes^34^. Here we present the first empirical measurements of microplastic deposition rates in flowing waters, providing a key contribution to the broader research encompassing microplastic ecology. We conclude that the retention of microplastic particles is influenced by a combination of density, particle shape, and biofilm colonization. By synthesizing deposition dynamics among different particle types, we show that microplastics are deposited in streams in a similar fashion as other well-studied particle types, thereby facilitating refinements in models of microplastic retention and export from streams and rivers.

### Effect of particle shape and biofilm colonization

Deposition rates varied by particle type, where shape, buoyancy, and biofilm presence explained differences among microplastic. Polypropylene pellets traveled the farthest, have the most uniform shape, lowest surface area to volume ratio, and the greatest buoyancy. Fragments showed the opposite pattern, with the shortest transport length, more complex and variable shape than pellets, and polystyrene density is greater than stream water. Given the size and bright colors of the plastic particles, we were able to directly observe the ‘behavior’ of pellets and fragments, in real time, during the experimental releases. Smooth-sided spheres moved farther than the sharp-sided fragments. We observed no benthic deposition due to the low density of polypropylene, and instead noticed retention of polypropylene pellets mostly in back waters and on stream edges. This observation suggested that pellets could be easily re-mobilized, and subsequently exported, via elevated flows. In contrast, polystyrene fragments were more commonly retained simply by sinking, and these dense fragments would probably resist mobilization during high flows to a greater degree than polypropylene pellets. Finally, both the v_dep_ of pellets and fragments increased when colonized by biofilms, which enhanced their relative ‘stickiness’.

The depositional patterns for fibers suggested controls on transport and deposition were different than for fragments and pellets. Fibers showed intermediate transport length, and a relatively small effect of biofilm colonization. We attribute these patterns to the difference in the shape of fibers compared to the other two particles. Acrylic fibers had relatively rigid, complex shapes that twisted in 3 dimensions. The long, thin fibers showed a tendency to be suspended in turbulent water relative to the simpler shape of fragments, which were retained more quickly, even though fragments were less dense than fibers. Biofilm colonization did not occur as readily on fibers compared to pellets and fragments, which may have been responsible for the relatively small influence of biofilms on fiber v_dep_. Overall, biofilms did not enhance fiber ‘stickiness’, and we infer that a primary factor controlling fiber deposition rates was their shape. Additional studies on microplastic transport with variable fiber dimensions could quantify this effect.

Microplastic pollution in aquatic ecosystems is a mixture of polymer types and densities, including buoyant (e.g., polypropylene and polyethylene), and non-buoyant (e.g., acrylic, polyvinyl chloride) compounds^28,43^. Particles of different densities that enter streams and rivers may be ‘sorted’, with the least dense being transported quickly downstream, while more dense particles sink, and may only be transported with elevated flows. Understanding the fate of microplastic particles over short and long time scales, and under variable flow conditions, is critical to measuring abundance in streams, and predicting the types of organisms most likely to encounter different microplastic polymers^13^.

### Predicting microplastic deposition velocity

When synthesizing patterns of deposition among particle types, particle diameter influences v_dep_ because larger particles can be more quickly trapped and retained compared to smaller ones. This pattern is useful when considering a broad range of particles, however, we note significant unexplained variation in the relationship between particle size and v_dep_, the driving role of CPOM, and the diversity of conditions under which the various v_dep_ measurements were collected (Fig. 2). Other studies have found similar trends. For example, Khatmullina and Isachenko *et al*. (2016) conducted direct measurements of v_fall_ for microplastic spheres, cylinders, and fibers, and used log-log plots to examine relationships between particle diameter and settling velocity. The authors showed that particle diameter was useful to predict microplastic v_fall_ rates when considering a large size gradient (0.5–5 mm); however, within a given size class, differences in particle shape and density generated significant variation in v_fall_. Williams *et al*.^42^ also found significant, positive relationships between ‘settling potential’ (analogous to v_dep_ in this study) of naturally occurring riverine sediment particles and particle size (range 2.5–500 µm). The authors showed differences in the slope of the relationship according to the sites the particles were collected from, and the particles’ capacity for flocculation. The composite data show that particle size is one of many components that affects deposition dynamics, and may be most informative when considering a relatively large gradient of particle diameters.

This analysis builds on previous work showing a significant relationship between v_dep_/v_fall_ relative to particle diameter to illustrate physical drivers of particle retention and transport in flowing waters^17^. Where the v_dep_/v_fall_ ratio is ~1, particles are retained in streams according to the expectations from gravity, which occurred for corn pollen and FPOM with diameter ~50–500 µm. For the smallest particles (i.e., <50 µm) including bacteria, yeast, and diatoms, the v_dep_/v_fall_ ratio was >1, suggesting that rapid deposition was driven by entrapment of particles by biofilms and interstitial spaces, rather than by sinking alone. For the particles with diameter >500 µm, including microplastic fragments and fibers, v_dep_/v_fall_ ratios were <1, indicating factors such as momentum and resuspension move particles downstream more than that expected by particle density alone.

One consideration for careful interpretation of the relationship between v_fall_/v_dep_ and particle size is the underlying relationships between v_fall_ and v_dep_ alone and particle diameter. When considered individually, rates of v_fall_ and v_dep_ from the data in Fig. 3 show a significant positive relationship with particle size (Supplemental Fig. 3), but with different slopes. Thus, the negative relationship between v_fall_/v_dep_ with particle size (Fig. 3) suggests a meaningful physical phenomenon. That is, the ratio of v_fall_ and v_dep_ varies according to particle size, and the factors which affect particle deposition in flowing waters can be interpreted according to the relative influence of gravity and particle momentum^17,44^.

Despite the pattern between v_fall_/v_dep_ and particle size, there is still unexplained variation in the relationship, which can be attributed to an array of factors that affect transport of naturally occurring particles in streams. Georgian *et al*.^17^ attributed some of the variance in the regression to difficulty in measuring and reporting particle diameter, which was estimated for some particles in the synthesis, particularly for filamentous and amorphous shapes^17^. Hamm *et al*.^44^ refer to the v_dep_/v_fall_ ratio as ‘dimensionless deposition velocity’ (W) and the authors quantified environmental factors driving W for glass microspheres (diameter 13–62 µm), including bed roughness, porosity, and turbulence^44^. Particle density is a key driver of deposition dynamics, and varies among materials included in Fig. 3. For example, Webster *et al*.^19^ report density of stream organic matter at 1.25 g/cm^3^, and the density of corn pollen, a surrogate for fine particulate organic matter, is 1.09 g/cm^3^ ^23,45^. Both are similar to the density of polystyrene fragments and acrylic fibers (1.04, and 1.17 g/cm^3^), but all are less dense than glass beads (2.48 g/cm^3^)^46^. Thus, the relationship between v_dep_/v_fall_ relative to particle size is useful when considering a relatively large size gradient and within individual size classes, but further assessments of factors intrinsic and extrinsic to the particles are needed to quantify and predict key drivers of particle deposition in streams.

There is a vast literature on particle transport which is available to guide future research on microplastic deposition, flocculation, bedload transport, and resuspension in flowing waters^19,42,44^. For example, this study was conducted at a single discharge and with only one type of sediment, and in artificial streams with little to no hyporheic exchange or suspended sediment. Previous research has used dynamic measurements of erosion, suspended sediment, and deposition to quantify the role of sediment flocculation, water velocity, and particle characteristics on sediment redistribution^44,47,48^. All of these factors will also affect microplastic movement and represent important factors to study for a greater understanding of their role in stream microplastic dynamics. Our results suggest that microplastic particle transport in flowing waters can be explained in part by well-established factors such as particle size, shape, and buoyancy. This study also shows the need for more research attention on the subject, as there is much to be learned about microplastic movement in streams and rivers by utilizing the full breadth of existing methods for quantifying particle dynamics.

We know of only one other study which attempted to measure microplastic v_dep_ *in situ*, and so we compared between studies to provide context for the magnitude of v_dep_ measured, and inform the ability to scale up microplastic v_dep_ from small streams to larger rivers. Hoellein *et al*.^12^ measured microplastic associated with a wastewater treatment plant (WWTP) on an urban river, sampling upstream of an effluent input, as well as several locations over 2 km downstream, where they found no significant decline in microplastic concentration in surface water. However, the authors explored potential v_dep_ rates based on the lack of measurable deposition over 2 km, concluding a maximum possible v_dep_ of 0.56 mm/s, and *potential* v_dep_ value of 0.11 mm/s (Table 3 in Hoellein *et al*.^12^). In this study, the mean (±standard deviation) v_dep_ across all 18 particle releases was 0.61 (±0.48) mm/s. Given that v_dep_ is designed as a comparative metric among systems of varying size, it is reassuring and instructive to find that the mean microplastic v_dep_ recorded in the small experimental stream studied here (Q = 1.4 L/s) compares favorably to the potential maximum v_dep_ measured *in situ* in a larger river (Q = 8,280 L/s). We acknowledge the limits of these comparisons, as these data represent the first two attempts to quantify v_dep_ for microplastic in flowing waters, yet the convergence of values for v_dep_ suggest predictable drivers on deposition. Nevertheless, further study is needed, along with more direct measurements of deposition, which will strengthen predictive models, including those that span a range of environmental conditions and particle types.

Empirical data such as the estimates made in this study can directly inform hydrologic models of microplastic transport within rivers. Current large-scale models of microplastic transport from its terrestrial and fluvial sources to oceans do not include a term for microplastic retention in streams and rivers^49,50^. Other models have based fluvial retention of microplastic on particle density and size^16,51^. For example, models of microplastic transport in Nizetto *et al*. (2016) assumed microplastic v_dep_ was equal to settling velocity (v_fall_ in this study; u_T_ in that study), so particles less dense than water exhibited no retention in rivers. The data presented here can contribute to refined model estimates by providing a direct v_dep_ measurement for polypropylene pellets in streams (density = 0.946 g/cm^3^), which was mediated not by sinking, but by retention at stream edges and in backwaters. In addition, our work shows that v_fall_ may be an appropriate surrogate for v_dep_ depending on size class; the values are most similar in the mid-range of diameters for published measurements (~50–500 µm), whereas v_fall_ is less representative of v_dep_ for other particle sizes. For small particles (<50 µm), v_fall_ underestimates v_dep_, while for larger particles (>500 µm), such as the microplastic particles used in this study, v_fall_ overestimates v_dep_. Comparisons suggest current models of microplastic movement underestimate microplastic retention in streams and rivers, to a potentially large degree. We suggest that the predictive ability of models can be improved by considering the ratio of v_dep_/v_fall_ based on particle diameter, combined with the v_dep_ rates presented for the lightweight polymers in this study.

### Additional factors affecting microplastic deposition in streams

While we show differences in deposition among particle types and with biofilm colonization status, many other key factors that influence microplastic retention in flowing waters should also be considered in future analyses, including the consideration of longer time scales, the potential for spatial variation in deposition rates and hydrodynamic conditions, the role of particle flocculation, and variation in particles’ physical properties. For example, the modified spiraling method used here only quantified short-term deposition, and it is unknown how long the microplastic particles remained in the locations at which they were initially retained. Particles may continue to move when agitated by other items in transport, or could be re-suspended by changes in water velocity. Particles in flowing waters are known to ‘jump’ or saltate along the benthic surface over a period of minutes, to hours, to days following their initial retention^52^. Longer term measurements of transport and retention are needed, and should include multiple time scales, as well as the addition of physiochemical variation, such as that occurring during storms. For example, in streams, seasonality influences temperature, organic matter inputs, riparian vegetation, and benthic biological community succession (i.e., presence of biofilms and submerged aquatic vegetation), all of which can affect microplastic deposition patterns.

Differences in microplastic retention should also be influenced by stream geomorphology, which can vary among stream reaches and from headwaters to larger rivers. The experimental stream used in this study was homogenous by design, for both substrate type and water velocity, yet even under these controlled conditions, there were occasional small backwater areas where we observed retention of polypropylene pellets. Mani *et al*. (2015) also suggested low flow areas, over short time periods, were sinks for microplastic particles in a longitudinal study of the Rhine River. Habitat features promoting depositional areas such as debris dams, meanders, and pools retain fine sediment^19,53^. We expect microplastic deposition to follow that of natural FPOM, with increased v_dep_ in low flow areas, and resuspension in high flow areas, but this has not yet been assessed. In addition, underlying substrate type, and the presence of periphyton and macrophytes may generate significant patchiness in benthic retention. More research is needed to quantify microplastic dynamics in streams using realistic assessments of factors that have been previously shown to influence particulate transport.

### Incorporating microplastic into paradigms of allochthonous carbon spiraling in streams

**Figure 4:**
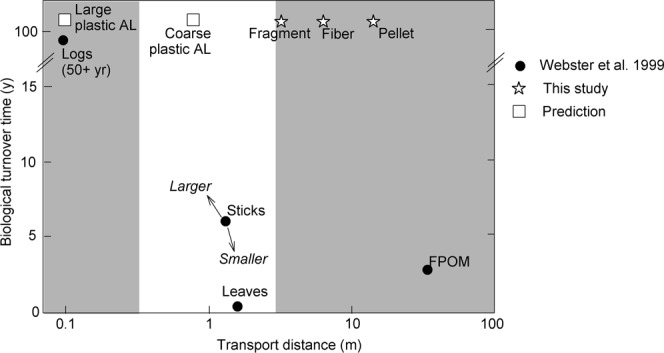
Transport distance and biological turnover time for naturally occurring allochthonous carbon including leaves, sticks, fine particulate organic matter (FPOM) and wood, originally synthesized in Webster *et al*.^19^. We added to this diagram by including direct measurements of transport distance for microplastic particles (i.e., fragment, pellet, and fibers), and an estimate of biological turnover time^54^. We also included predictions of transport distance for coarse plastic anthropogenic litter (AL, i.e., shopping bags, bottles, and food wrappers), and large plastic AL (i.e., construction debris, shopping carts).

Webster *et al*.^19^ synthesized an abundance of research on particle transport and biological breakdown of allochthonous organic material entering streams at Coweeta Hydrologic Lab in North Carolina, USA. The synthesis showed the relative influence of hydrology and biology on downstream transport of FPOM, leaf litter, small sticks, and large woody debris. Plastic pollution represents a novel allochthonous carbon input to streams, and can be directly compared to naturally occurring materials. We combined our measurements of transport length (as S_w_) for microplastic particles with data from Webster *et al*.^19^, and results show that microplastic transport distance falls between FPOM and leaf litter (Fig. 4).

Given its resistance to biological breakdown, plastic litter is a long-term component of aquatic ecosystems relative to naturally occurring allochthonous carbon^1^. Biological degradation of plastic varies with environmental conditions and polymer types. For example, the biodegradation rate of polyethylene was measured in laboratory conditions, with an estimated loss rate of 0.1% per year, or a turnover time of 1000 years^54^. Physical factors such as ultraviolet light and abrasion enhance biological breakdown^55^. While the expanded synthesis in Fig. 4 illustrates that microplastic particles are transported in a similar fashion to naturally occurring particles, the lack of biodegradation for plastics suggests an individual particle may complete many ‘spirals’ downstream prior to permanent removal. Finally, we predicted transport distance and biological turnover for larger plastic anthropogenic litter (AL) types common in urban streams. We speculate that the movement of ‘coarse’ plastic AL (e.g., shopping bags, plastic bottles, and food wrappers^5^) will be similar to leaves and sticks, and that large plastic AL (e.g., shopping carts, construction debris, and pipes) will move like large woody debris (Fig. 4). These empirical measurements have not yet been made, but this conceptual framework can be used to develop hypotheses regarding the transport of plastic and naturally occurring particulate matter of all size classes in streams.

## Conclusion

Plastic pollution is a global, pervasive, increasing, and permanent feature of present day aquatic ecosystems^55^. While research on microplastic abundance, movement, fate, and biological interactions in freshwaters are less common than oceans, the body of literature for freshwater ecosystems is growing. Measuring and predicting the transport and retention of plastic particles in streams and rivers is a critical knowledge gap in this field of study. More empirical studies are needed to account for the influences of material type, environmental conditions, and particle dimensions on its movement. As the field grows, foundational methods and paradigms from stream ecosystem ecology should be adapted for research on the ecology of plastic particles, which will inform robust assessments of plastic ecology in flowing waters, and thereby contributing to global models and budgets of plastic pollution.

## Supplementary information

Supplemental material

## Data Availability

Data generated or analyzed during this study are included in this published article (and its Supplementary Information files). Further details on datasets generated during and/or analyzed during the current study are available from the corresponding author on reasonable request.
