# Supplementary material for: Microplastic deposition velocity in streams follows patterns for naturally occurring allochthonous particles
Source: Sci Rep. 2019 Mar 6;9:3740. doi: 10.1038/s41598-019-40126-3 (PMC6403300; doi:10.1038/s41598-019-40126-3)
Supplement: Supplementary file 1 — Supplemental material [file 41598_2019_40126_MOESM1_ESM.pdf]

Supplemental Information

Microplastic deposition velocity in streams follows patterns for naturally occurring allochthonous particles

Timothy J. Hoellein[1*], Arial J. Shogren[2,3], Jennifer L. Tank[2], Paul Risteca[1] John J. Kelly[1]

[1]Dept. Biology, Loyola Univ. Chicago, 1032 W Sheridan Rd, Chicago IL, USA 60660.

[2]Dept. Biological Sciences, Univ. of Notre Dame, Notre Dame, IN, USA 46556

[3]*Current address*: Dept. Earth and Env. Sci., Michigan State Univ., 306 Natural Sci. Bldg. East Lansing, MI 48824

**Corresponding author*. Phone: 773-508-8859, Fax: 773-508-5636, email: thoellein@luc.edu

Supplemental Table 1. Physical characteristics of the three study reaches of the experimental stream. Discharge (Q), width (w), depth (z), water velocity (V), and slope (S) were measured. Hydraulic radius (R) = (w*z)/w, shear stress = ρgRS (where ρ = density of water at 10°C, g = gravitational constant), Reynolds number (Re) = (V*z)/*v* (where *v*=kinematic viscosity of water at 10°C), and Froude number = $V/[(g*z)^{0.5}]$.

	Reach 1	Reach 2	Reach 3
discharge (L/s)	1.45	1.43	1.35
width (m)	0.458	0.460	0.462
depth (m)	0.039	0.036	0.033
velocity (m/s)	0.082	0.087	0.090
hydraulic radius (m)	0.039	0.036	0.033
slope (m/m)	0.010	0.010	0.010
shear stress (N/m^2)	3.70	3.41	3.12
Reynolds number	2435	2389	2248
Froude number	0.134	0.148	0.159

Supplemental Table 2. Meta-analysis of particle deposition velocity (v_{dep}) and diameter in streams. The * in the diameter column indicates longest axis rather than diameter which was used for the last 4 particles on the table. Abbreviations: LFPOM = large fine particulate organic matter, MFPOM = medium fine particulate organic matter. SFPOM = small fine particulate organic matter, FPOM = fine particulate organic matter, VFPOM, very fine particulate organic matter, UFPOM = ultra-fine particulate organic matter.

Particle type	Diameter (µm)	vdep (mm/s)	Citation
LFPOM	280	4.3	Kazmierczak et al. 1987
MFPOM	105	2.8	Kazmierczak et al. 1987
SFPOM	43	1.7	Kazmierczak et al. 1987
FPOM	24	0.8	Kazmierczak et al. 1987
VFPOM	10	0.7	Kazmierczak et al. 1987
UFPOM	0.05	0.6	Kazmierczak et al. 1987
FPOM	77.5	1.7	Cushing et al. 1993
FPOM	77.5	1.0	Cushing et al. 1993
FPOM	77.5	1.8	Cushing et al. 1993
Yeast	5.8	0.031	Paul & Hall 2002
Bacteria	2.0	0.093	Hall et al. 1996
VFPOM	26.7	0.031	Hunken and Mutz 2007
VFPOM	26.7	0.111	Hunken and Mutz 2007
VFPOM	26.7	0.231	Hunken and Mutz 2007
VFPOM	26.7	0.075	Hunken and Mutz 2007
VFPOM	26.7	0.153	Hunken and Mutz 2007
VFPOM	26.7	0.053	Hunken and Mutz 2007
VFPOM	26.7	0.253	Hunken and Mutz 2007
VFPOM	26.7	0.242	Hunken and Mutz 2007
VFPOM	26.7	0.078	Hunken and Mutz 2007
VFPOM	26.7	0.039	Hunken and Mutz 2007
VFPOM	26.7	0.036	Hunken and Mutz 2007
VFPOM	26.7	0.094	Hunken and Mutz 2007
VFPOM	26.7	0.186	Hunken and Mutz 2007
VFPOM	26.7	0.342	Hunken and Mutz 2007
VFPOM	26.7	0.114	Hunken and Mutz 2007
VFPOM	26.7	0.069	Hunken and Mutz 2007
VFPOM	26.7	0.067	Hunken and Mutz 2007

VFPOM	26.7	0.100	Hunken and Mutz 2007
Pollen	87	0.311	Miller & Georgian 1992
Pollen	87	0.305	Miller & Georgian 1992
Pollen	87	0.263	Miller & Georgian 1992
Pollen	87	0.313	Georgian et al. 2003
Pollen	87	0.146	Georgian et al. 2003
Pollen	87	0.094	Georgian et al. 2003
Pollen	87	0.12	Georgian et al. 2003
FPOM	53	0.244	Georgian et al. 2003
FPOM	53	0.16	Georgian et al. 2003
FPOM	53	0.0514	Georgian et al. 2003
FPOM	53	0.25	Georgian et al. 2003
FPOM	53	0.052	Newbold et al. 2005
FPOM	53	0.03	Newbold et al. 2005
FPOM	53	0.15	Newbold et al. 2005
VFPOM	15	0.047	Newbold et al. 2005
VFPOM	15	0.041	Newbold et al. 2005
VFPOM	15	0.078	Newbold et al. 2005
Glass Beads	78	0.643	Ehrman et al. 1994
Pollen	87	1.26	Ehrman et al. 1994
FPOM	53	0.87	Minshall et al 2000
FPOM	53	0.24	Minshall et al 2000
FPOM	53	0.17	Minshall et al 2000
FPOM	53	0.06	Minshall et al 2000
FPOM	53	0.26	Minshall et al 2000
FPOM	53	0.83	Minshall et al 2000
FPOM	53	0.14	Minshall et al 2000
FPOM	53	0.07	Minshall et al 2000
FPOM	53	1.1	Thomas et al. 2001
FPOM	53	0.24	Thomas et al. 2001
VFPOM	15	1.09	Thomas et al. 2001
VFPOM	15	0.16	Thomas et al. 2001
MPOM	107	0.53	Thomas et al. 2001
Diatoms	15	0.99	Thomas et al. 2001
FPOM	53	0.63	Thomas et al. 2001
FPOM	53	1.1	Thomas et al. 2001
FPOM	53	0.24	Thomas et al. 2001
FPOM	53	0.17	Thomas et al. 2001
FPOM	53	0.34	Thomas et al. 2001
FPOM	53	1.03	Thomas et al. 2001
FPOM	53	0.62	Thomas et al. 2001

VFPOM	15	0.43	Thomas et al. 2001
VFPOM	15	1.09	Thomas et al. 2001
VFPOM	15	0.16	Thomas et al. 2001
VFPOM	15	0.11	Thomas et al. 2001
VFPOM	15	0.13	Thomas et al. 2001
VFPOM	15	0.42	Thomas et al. 2001
VFPOM	15	0.43	Thomas et al. 2001
Spores	42	0.05	Wanner & Pusch 2000
Yeast	7	0.08	Shogren et al. (unpub data)
Yeast	7	0.04	Shogren et al. (unpub data)
Yeast	7	0.01	Shogren et al. (unpub data)
Pollen	70	0.12	Shogren et al. (unpub data)
Pollen	70	0.44	Shogren et al. (unpub data)
Pollen	70	0.4	Shogren et al. (unpub data)
Pollen	70	0.23	Shogren et al. (unpub data)
Pollen	54	0.313	Webster et al. 1999
Glass beads	78	3.97	Webster et al. 1999
FPOM	43	1.19	Webster et al. 1988
Pellets	3,000	0.28	This study
Fragment	1,500	1.08	This study
Fiber	1,500*	0.46	This study
Stick	460,000*	3.784	Cordova et al. 2008
Pine needle	100,000*	5.858	Cordova et al. 2008
G. biloba leaf	53,000*	4.016	Cordova et al. 2008

Supplemental Table 3. Results for the deposition measurements for all of the particle releases conducted during the study. Abbreviations: S_w = transport length, v_{dep} = deposition velocity, v_{fall} = sinking velocity, and N/A = not applicable because v_{fall} = 0 for polypropylene pellets.

	Particle	biofilm	Slope	R^2	S_w (m)	v_{dep} (mm/s)	v_{dep}/v_{fall}
Reach 1							
Release 1	Pellet	yes	-0.091	1.00	11.0	0.277	*(N/A)*
Release 2	Fragment	yes	-0.310	1.00	3.2	0.948	0.061
Release 3	Fiber	yes	-0.122	0.99	8.2	0.372	0.030
Release 4	Pellet	no	-0.026	1.00	38.3	0.085	*(N/A)*
Release 5	Fragment	no	-0.152	0.94	6.6	0.497	0.032
Release 6	Fiber	no	-0.190	0.99	5.3	0.624	0.050
Reach 2							
Release 7	Pellet	yes	-0.155	1.00	6.5	0.452	*(N/A)*
Release 8	Fragment	yes	-0.760	1.00	1.3	2.220	0.142
Release 9	Fiber	yes	-0.181	0.97	5.5	0.530	0.042
Release 10	Pellet	no	-0.070	1.00	14.4	0.203	*(N/A)*
Release 11	Fragment	no	-0.273	1.00	3.7	0.797	0.051
Release 12	Fiber	no	-0.140	0.93	7.2	0.408	0.033
Reach 3							
Release 13	Pellet	yes	-0.143	1.00	7.0	0.375	*(N/A)*
Release 14	Fragment	yes	-0.375	0.93	2.7	0.986	0.063
Release 15	Fiber	yes	-0.141	0.97	7.1	0.371	0.030
Release 16	Pellet	no	-0.087	0.98	11.5	0.282	*(N/A)*
Release 17	Fragment	no	-0.310	0.83	3.2	1.008	0.065
Release 18	Fiber	no	-0.144	1.00	6.9	0.468	0.037

Supplemental Figure legends.

Supplemental Figure 1. Image of particle transport in the experimental stream. This image was taken 40 seconds after a pellet release, 10 m downstream of the release point. These images were collected every 5 seconds and were used to measure particle transport as described in methods.

Supplemental Figure 2. Particle counts for microplastic transport at collection sites located 5, 10, and 15 m from the particle release location, according to time since particle released. (A) pellets, (B) fragments, and (C) fibers. Transport length is calculated from the slope of the regression line, generated from the conservative tracer (conductivity) and microplastic that passed by distance from the microplastic release point (i.e., natural log of microplastic/conductivity), (D) pellets, (E) fragments, and (F) fibers.

Supplemental Figure 3. The synthesis of particle transport first developed by Georgian et al. (2003) showing the relationship between (A) settling velocity (v_{fall}) and (B) deposition velocity (v_{dep}) relative to particle diameter (log transformed), for data collected in flowing waters. We added data for microplastic fragments and fibers collected in this study (gray), as well as results from Hunken and Mutz (2007) and Cushing et al. (1993). In (A), v_{fall} for fibers and fragments was presented only for non-biofilm colonized particles. In (B), v_{dep} was measured for microplastic with and without biofilm colonization. Results for linear regression between log-transformed axes are shown in the upper left of each panel.

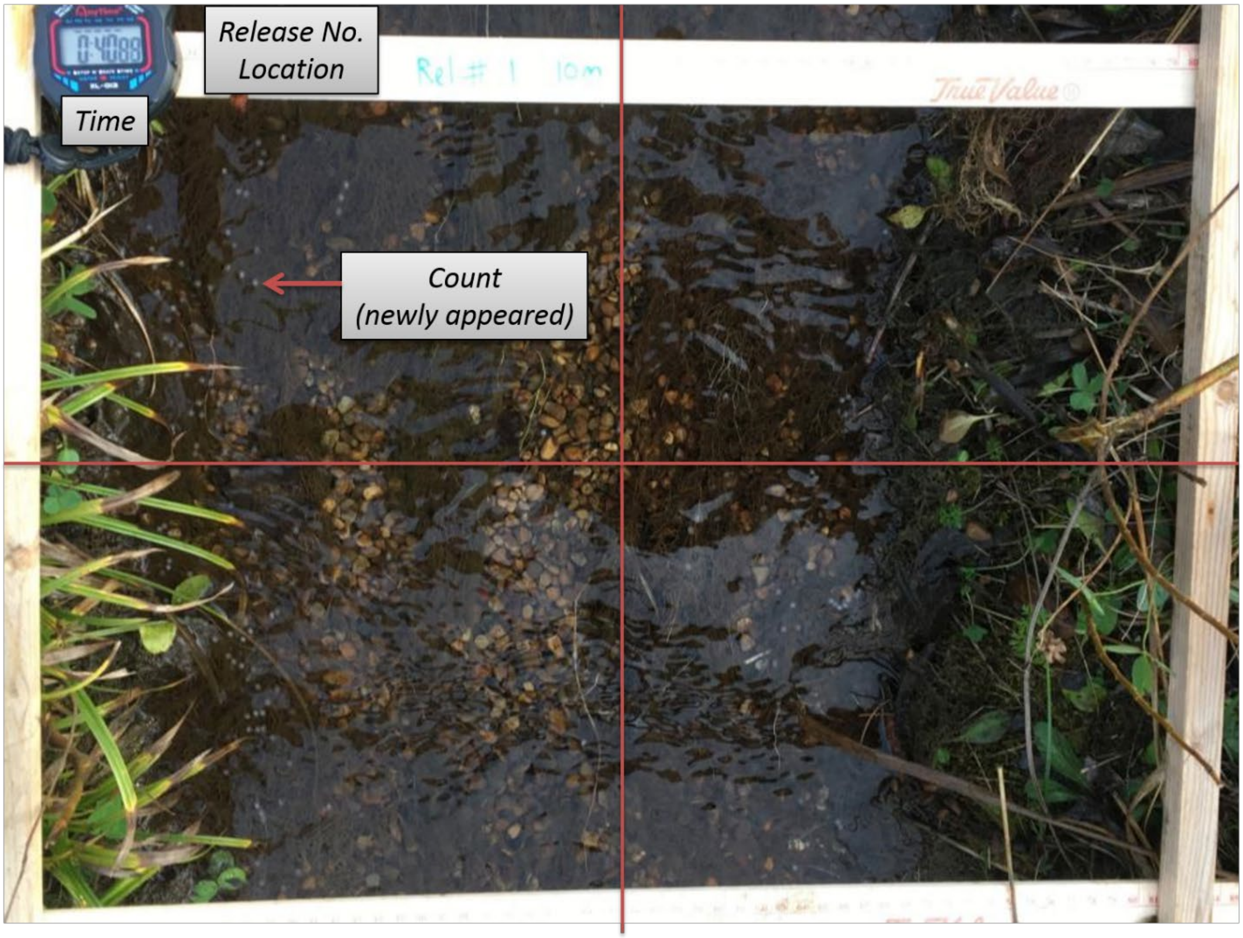

Supplemental Figure 1

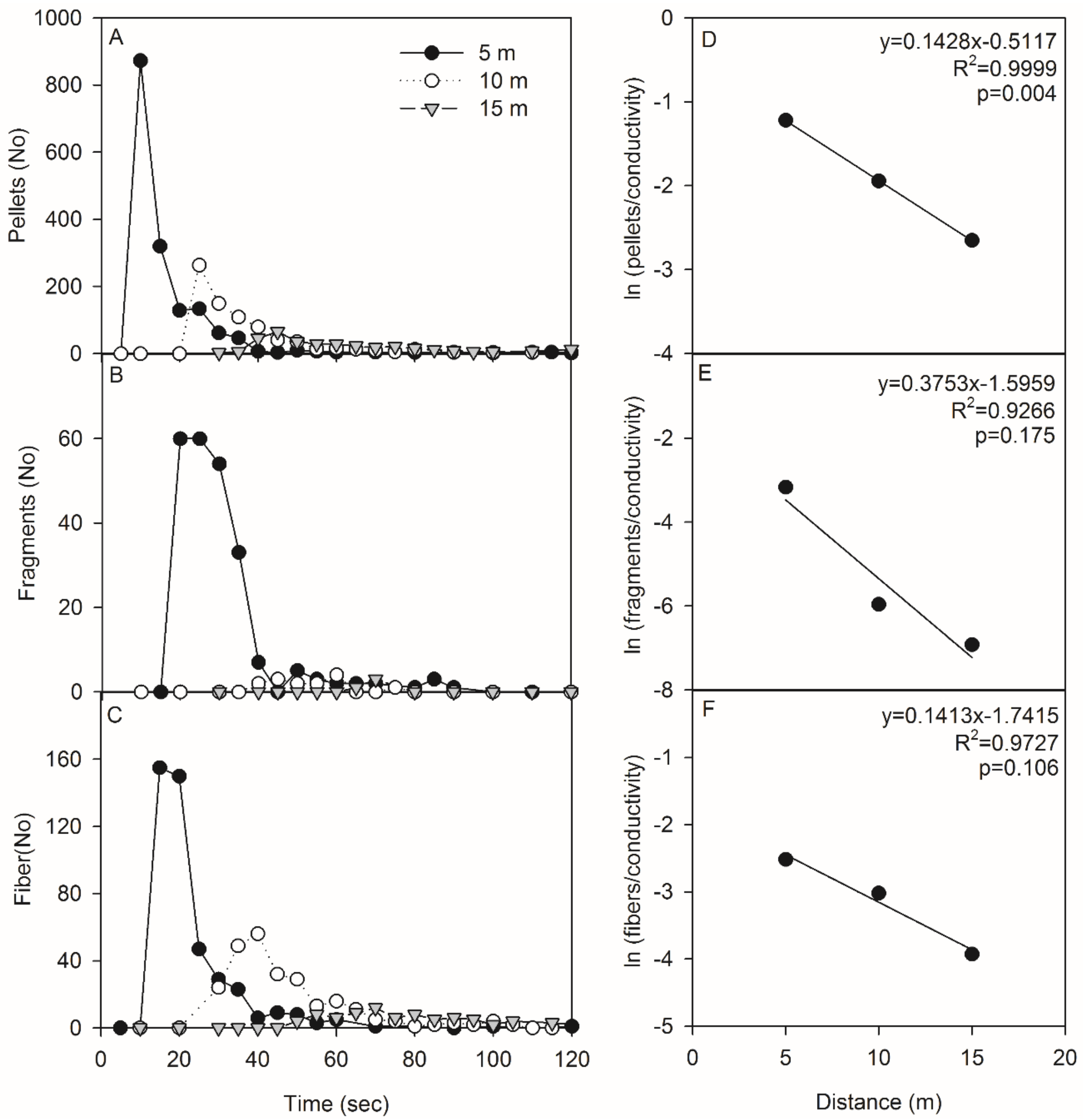

Supplemental Figure 2

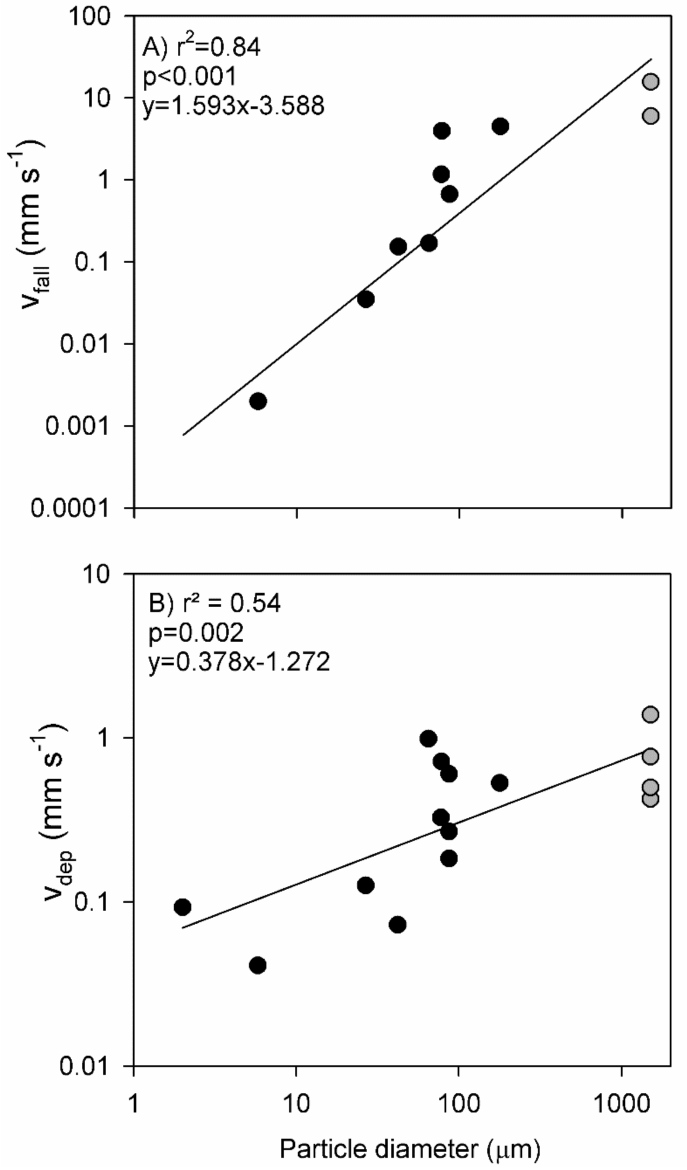

Supplemental Figure 3
